# Socio-Economic Inequalities, Out-of-Pocket Payment and Consumers' Satisfaction with Primary Health Care: Data from the National Adult Consumers' Satisfaction Survey in Serbia 2009–2015

**DOI:** 10.3389/fphar.2017.00147

**Published:** 2017-03-28

**Authors:** Katarina Vojvodic, Zorica Terzic-Supic, Milena Santric-Milicevic, Gert W. Wolf

**Affiliations:** ^1^Institute of Public Health of BelgradeBelgrade, Serbia; ^2^School of Medicine, Institute of Social Medicine, University of BelgradeBelgrade, Serbia; ^3^Department of Geography, University of KlagenfurtKlagenfurt, Austria

**Keywords:** consumers' satisfaction, primary health care, unmet needs, health, payment

## Consumers' satisfaction and health care quality

For over 30 years, a movement for putting primary health care (PHC) in the driver's seat of the health care system has aimed at promoting values such as equity, patient-centeredness, community participation and self-determination (Saltman et al., 2006). While at the beginning these values were considered radical, they are now widely accepted in health care (World Health Organization, 2008). This paradigm shift becomes also obvious when WHO adopted Millennium Development Goals, whose achievements were most thoroughly studied by the Global Burden of Disease Project (Lim et al., 2016). As was shown by different authors, the efficiency of PHC depends primarily on factors, such as the historical background, the occurrence of health problems, the characteristics of the health system as well as the social features of consumers (Ros et al., 2000; Walshe and Freeman, 2002).

One of the characteristics of a good health care system is its capability to satisfy consumers' needs (Sándor et al., 2016). Unmet health care needs depend primarily on consumers' socio-demographic characteristics, their experience with health care services, the quality of the offered health care and the level of consumers' health literacy (Chaupain-Guillot and Guillot, 2015). The main reasons for unmet heath care needs are cost, distance, waiting lists, the lack of cultural sensitivities and discrimination (Eurostat, 2016). In the EU-28, the most common reason, affecting about one third of all reported cases and involving 2.4% of the population, for not having a medical examination or treatment were excessive high costs (Eurostat, 2016).

Satisfaction of customers/patients does not only map positive assessment to the different dimensions of health care (Linder-Pelz, 1982), but also represents an important quality indicator (Donabedian, 2003). Patients' satisfaction is regarded as a crucial issue with respect to the creation of a high quality, safe and effective health care system (Tucker, 2002; National Health Service Corporation, 2010; Hinchcliff et al., 2014). Satisfied patients are more willing to undergo recommended therapies, thus entailing better health outcomes including a lower mortality rate (Chue, 2006; Glickman et al., 2010).

Patients' satisfaction includes on the one hand the nature of the first contact with the health system *per se* and on the other hand the staff's interaction with patients (National Health Service Corporation, 2010). Apart from the strong relationship between patients' satisfaction and the structure and processes of the health care system (Rademakers et al., 2011), socio-demographic characteristics have also a significant influence on patients' satisfaction with respect to health care (Rahmqvist and Bara, 2010). Female and older patients, patients with a high school or university diploma or patients with a very good perceived household well-being (HWB) were more likely to be satisfied with health care than other patients (Kontopantelis et al., 2010; Davey et al., 2013).

Great efforts were undertaken in the past with respect to the development of instruments for measuring patients' satisfaction (Garratt et al., 2008). In Serbia, policy makers established the mechanisms for a sustainable continuous improvement of the health care system (Simic et al., 2010) including the continuing education of the general practitioners (GPs) (Santric Milicevic et al., 2011), the supply of drugs (Jakovljevic et al., 2015) and financing (Simic et al., 2010). In addition, the Serbian managers were educated how to apply knowledge to utilize the results for further quality improvement (Terzic Supic et al., 2010; Santric Milicevic et al., 2011).

## Population development and health care expenditure in Serbia 2009–2015

Between 2009 and 2015, the population of Serbia decreased from 7,321,000 to 7,095,000, i.e., about 220,000 or 3.1%. In contrast, the proportion of the age group 65+ increased from 17.2 to 18.3% (World Health Organization, 2015).

In this 7-year period the Gross Domestic Product (GDP) enlarged between 2009 and 2013 from $5,821.30 to $6,353.80 per capita, decreased in 2014 to $6,200.17 per capita, only to reach its minimum in 2015 with $5,144.00 per capita. Total health expenditure as a percentage of GDP increased from 9.9% in 2009 to 10.4% in 2014 (the data for 2015 are not yet available), while in the same period public sector expenditures for health as a percentage of GDP varied between 6.1% in 2009 and 6.4% in 2014 (World Health Organization, 2015). A relationship between health expenditures per capita and longevity was revealed for the vast majority of European countries, notwithstanding they are members of the EU or not (Jakovljevic et al., 2016a).

Fenton et al. (2012) reported that higher patients' satisfaction is positive correlated with both total health care expenditure and expenditure on prescribed drugs. In contrast, political interventions with the objective of cost reduction have a negative impact on the perception of all health care professionals with respect to health care quality (Jakovljevic et al., 2016b). As regards the Serbian health care system, it is essential not only to consider the health care expenditure, but also existing socioeconomic inequities (Jankovic and Simic, 2012; Radevic et al., 2016) and the burden of disease (Sipetic et al., 2013).

## Primary health care service in Serbia 2009–2015

Public PHC is provided in Serbia on municipality level by PHC centers, which are established in almost all municipalities according to the Ministry of Health of Serbia Decree on the Health Care Institutions Network Plan (Ministry of Health of the Republic of Serbia, 2006). These health care centers provide a wide range of preventive and curative health care for all demographic groups and represent the places of first contact with the public health care system in Serbia (Simic et al., 2010). In Serbia, patients are required to register with one physician of their choice, representing the doctor of first contact with the health care system. The chosen doctor for the adult population is a medical doctor or a general practitioner (GP) specialist.

Health care in Serbia is financed primarily by mandatory contributions to the National Health Insurance Fund, whereby almost one quarter (24.6%) of the entire health budget is spent on PHC. Mandatory health insurance premiums are levied on the salaries of the employees, farmers and self-employed. Another source of financing is out-of-pocket payments for health care services and drugs (participation/official co-payments or full price). Between 2009 and 2015, the out-of-pocket payments as proportion of total health expenditure rose from 35.2 to 36.6% (World Health Organization, 2015). Though official co-payments represent a significant share of out-of-pocket payments in Serbia (Arsenijevic et al., 2014), they are much lower as compared with some neighboring countries (Atanasova et al., 2013). Co-payments for GP visits, specialist visits, diagnostic procedures and drugs are mandatory for all patients, with exceptions for children, pregnant woman, people who suffer from some chronic non-communicable diseases, disabilities, for members of the Roma population etc. (Ministry of Health of the Republic of Serbia, 2005).

Summarizing, it can be said that the Serbian health care policy has changed significantly over the past years toward patient's centeredness and quality improvement (Simic et al., 2010), which is well documented in different government papers (Kosanovic and Andelski, 2015) and by the Health Consumer Index (Health Consumer Powerhouse, 2017).

## The Serbian health care consumers' satisfaction surveys between 2009 and 2015

Consumers' satisfaction surveys were conducted from 2009 to 2015 in all public PHCs in Serbia according to the methodology provided by the Institute of Public Health of Serbia “Dr. Milan Jovanovic Batut”. The data were collected by using a questionnaire that was designed according to the WHO's recommendation for estimating availability, utilization, coordination and comprehensiveness of a health care system and that was, in addition, adopted with respect to the Serbian PHC system. It referred to socio-demographic characteristics (gender, age, the level of education, perceived HWB), received preventive care counseling, level of satisfaction (with PHC services, GPs, nurses, equipment), utilization, official co-payments (for GP visits, visits to specialists and drugs) and overall satisfaction with the offered PHC services.

The data for the present study were gathered in the course of cross sectional surveys conducted in 158 PHC centers in Serbia, among adult patients who visited their GPs during the working hours between 7:00 and 20:00. The periods of the studies were the same for all PHC centers. Participation was voluntarily and all respondents were informed about the purpose of the study and they could quit the interview at any time. Anonymity, confidentiality and privacy of data were explained and guaranteed (name, address, phone number or other personal data were not collected). The participants were asked to answer the questions after having visited their GPs. In the period between 2009 and 2015 an overall of 206,088 patients were included in the surveys, thus yielding annual response rates of more than 70%.

In the present study demographic characteristics, such as age or gender, the socioeconomic status comprising education or the perceived HWB, the out-of-pocket payments for visiting GPs and specialists as well as for prescribed drugs were analyzed in combination with the patients' satisfaction with the offered PHC services. For this purpose, the five-grade Likert scale (used to measure consumers' satisfaction) was modified into a three grade version running “satisfied,” “neither satisfied nor dissatisfied” and “dissatisfied”. Patients' unmet needs were also analyzed in order to find out if necessary visits to a GP were avoided or postponed due to the inability to pay for them.

The data set was uploaded to a public repository *Figshare* and is available at https://figshare.com/s/bb621ab20afb39b611e3. Data were uploaded as an Excel file, the questionnaire as pdf-file.

## Consumers' satisfaction surveys: data analysis between 2009 and 2015

### Socio-economic characteristics of the respondents

Among the respondents, there were more women (55.3%) than men (44.7%). The highest percentage of respondents was in the age class between 50 and 59 (22.2%). The average age was 51.2 ± 15.7, whereby male patients were older (52.1 ± 15.8) than female ones (50.5 ± 15.5). The majority of respondents had attended a secondary school (53.9%), while 6.2% had less than primary school. A statistically significant difference between the levels of education with respect to gender could be observed (*p* < 0.05). Women attended more frequently only a primary school (m: 18.7%, f: 19.5%) or even less (m: 5.5%, f: 6.6%), while men attended more frequently a secondary school (m: 54.8%, f: 53.3%) or had a high school or university diploma (m: 20.9%, f: 20.6%). Most of the respondents, regardless of gender, perceived their HWB as medium (47.4%), while only a minority perceived it as very good (4.4%). There was a statistically significant difference of the perceived HWB with respect to gender (*p* < 0.05). Men categorized their HWB more frequently as bad than women (m: 14.9%, f: 14.0%), very bad (m: 7.0%, f: 6.1%), but also as very good (m: 4.5%, f: 4.3%).

### Consumers' satisfaction with public health care

More than three quarters of the patients (79.0%) were satisfied with public health care. There was a statistically significant difference between the numbers of satisfied patients with respect to the year of the Health Care Consumers' Satisfaction Survey. The largest proportion of satisfied patients was observed in 2010 with 83.1%, while the largest proportion of dissatisfied was found in 2014 with 8.5%. Apart from this, there was a statistically significant difference between the numbers of satisfied patients with PHC services with respect to gender, the education level and the perceived HWB (*p* < 0.05). Female consumers were more satisfied (79.1%) as well as older ones (81.1% for the age group 80+, 80.8% for the age group 60–69). Apart from the two previously mentioned age groups, the most satisfied patients were those with a high school or university diploma (80.0%) and those with a very good perceived HWB (84.9%). In contrast, the group of the most dissatisfied patients comprised the youngest (7.6%), people with an education level less than primary school (9.0%) and with very bad perceived HWB (13.9%). Consumers' satisfaction with PHC services by the year of survey and selected socio-demographic characteristics is shown in Table 1.

**Table 1:** **Contingency table of consumers' satisfaction with PHC services by the year of survey and selected socio-demographic characteristics**.

**Variables**		**Dissatisfied**		**Neither satisfied nor satisfied**		**Satisfied**		***p***
		***N***	**%**	***N***	**%**	***N***	**%**	
Year	2009	1.698	5.7	4.277	142	24.072	80.1	<0.05
	2010	1.463	4.8	3.726	12.1	25.510	83.1	
	2011	1.827	6.1	3.872	13.0	24.168	80.9	
	2012	2.111	7.7	4.122	15.0	21.336	77.4	
	2013	2.162	7.7	4.192	14.9	21.701	77.4	
	2014	2.129	8.5	4.079	16.3	18.892	75.3	
	2015	2.101	7.4	4.297	15.1	22.122	77.6	
Gender	Male	6.154	7.1	12.207	14.0	68.784	78.9	<0.05
	Female	6.916	6.4	15.726	14.5	85.731	79.1	
Age	19 through 29	1.491	7.6	3.430	17.6	14.616	74.8	<0.05
	30 through 39	2.240	7.0	5.304	16.7	24.234	76.3	
	40 through 49	2.581	6.6	5.877	15.1	30.408	78.2	
	50 through 59	3.023	6.8	5.906	13.3	35.430	79.9	
	60 through 69	2.419	6.6	4.670	12.7	29.824	80.8	
	70 through 79	1.331	5.9	2.652	11.8	18.445	52.2	
	80 and more	406	6.8	726	12.1	4.844	81.1	
Education	Less than primary school	1.084	9.0	1.646	13.5	9.448	77.6	<0.05
	Primary school	2.561	6.7	5.549	14.5	30.056	78.8	
	Secondary school	6.926	6.5	15.764	14.7	84.421	78.8	
	High school or university	2.809	6.8	5.449	13.2	32.984	80.0	
Household well-being	Very bad	1.789	13.9	2.376	18.5	8.701	67.6	<0.05
	Bad	2.275	8.0	5.200	18.2	21.116	73.9	
	Medium	5.862	6.2	14.242	15.1	74.212	78.7	
	Good	2.926	5.4	5.872	10.8	45.625	83.8	
	Very good	560	6.3	774	8.8	7.508	84.9	

### Out-of-pocket payment for health services

The majority of respondents (54.5%) did not have to pay for GP visits, but 42.5% had to pay a co-payment. Approximately a half (51.1%) had to pay their co-payment for drugs, 40.4% got drugs free of charge and 2.9% had to pay the full price, while 5.6% did not know the answer. Since 2009 the number of those who did not have to pay for GP visits increased by 10% and the number of those who did not have to pay for prescribed drugs increased by 7%. Most of the patients, who did not have to pay for GP visits, got also free of charge drugs (72.6%), while only a small portion had to pay participation (21.1%). About half of the respondents thought that visits to specialists (with GP's referral) were free of charge 45.9%, while 44.7% thought that they have to pay a co-payment.

Throughout the research period 74.3% of the respondents did not avoid or postpone a GP visit, because they could not pay for it or for drugs, respectively; only a minority of 13.6% avoided or postponed such a visit, while 12.1% could not remember. There was a statistically significant difference regarding the number of respondents who avoided or postponed GP visits with respect to different socio-demographic characteristics (*p* < 0.05). Avoiding and postponing was more frequent among female respondents (13.9%), patients in the age group from 50 to 59 (14.6%), respondents with an education less than primary school (20.7%) and among those patients who perceived their HWB as bad or very bad (together 32.0%).

### Consumers' satisfaction, payment for health care services and unmet needs

There was a statistically significant difference between the number of satisfied consumers with respect to payment or not for PHC services (*p* < 0.05). Satisfied consumers were those who paid participation for GP visits (80.2%) or got it for free of charge (80.0%), got drugs for free of charge (82.2%) or thought visits to a specialist (with GP referral) were free of charge (82.0%). The most dissatisfied patients were those who got GP visits free of charge (7.2%), got drugs for free (5.0%), but had a co-payment for specialists' visits (4.9%) or missed or postponed visit to GP (5.3%). Consumers' satisfaction with PHC services by the cost of service and unmet needs is shown in Table 2.

**Table 2:** **Contingency table of patients' satisfaction with PHC services by cost of services and unmet needs**.

**Variables**		**Dissatisfied**		**Neither dissatisfied nor satisfied**		**Satisfied**		***P***
		***N***	**%**	***N***	**%**	***N***	**%**	
Is today's visit to GP for free or not?	Free	7.557	7.2	13.474	12.8	84.092	80.0	<0.05
	Participation	4.122	5.0	12.137	14.8	65.860	80.2	
	Full price	304	25.8	313	26.5	562	47.7	
	I don't know	589	12.7	1.394	30.0	2.664	57.3	
Are today's prescribed drugs for free or not?	Free	4.402	6.2	8.225	11.6	58.394	82.2	<0.05
	Participation	4.512	5.0	12.831	14.2	72.747	80.7	
	Full price	794	15.7	1.468	29.1	2.790	55.2	
	I don't know	886	9.0	2.637	26.7	6.362	64.4	
Is the visit to a specialist for free or not?	Free	4.830	6.1	9.432	11.9	64.817	82.0	<0.05
	Participation	3.761	4.9	11.050	14.4	62.148	80.8	
	Full price	669	18.3	990	27.1	1.994	54.6	
	I don't know	1.006	8.1	3.119	25.0	8.362	67.0	
Have you in the last 12 months missed or postponed visit to the GP because you have to pay for it?	Yes	4.108	15.4	6.409	24.0	16.238	60.7	<0.05
	No	7.731	5.3	16.902	11.5	122.087	83.2	
	I don't remember	1.375	5.8	4.824	20.2	17.647	74.0	

There was a statistically significant difference between the number of consumers who were dissatisfied and consumers with unmet needs due to the inability to pay for health care services in the previous 12 months. As shown in Table 2, consumers who experienced unmet needs were dissatisfied in a much greater number (15.4%) than those without such an experience (5.3%).

### Prediction of the number of dissatisfied consumers for the period 2016–2018

The temporal development of the number of those consumers who were either dissatisfied or very dissatisfied with the Serbian PHC system was analyzed. A first inspection of the data revealed that their percentage has continuously grown over the past years. The trend can thereby be described by the regression line y = 0.0051x – 10.113, with the corresponding Pearson correlation coefficient *r* = 0.821 (*p* < 0.05) indicating a strong linear relationship between these two variables. Based on this regression analysis, an attempt was undertaken to predict the percentage of those consumers that will be dissatisfied or very dissatisfied with the Serbian PHC system within the next 3 years. According to the respective calculations, the percentage of dissatisfied or very dissatisfied patients will increase in 2016 to 8.85% (with the 95% prediction interval ranging from 6.05 to 11.64%), in 2017 to 9.35% (with the 95% prediction interval ranging from 6.30 to 12.40%) and, finally, in 2018 to 9.86% (with the 95% prediction interval ranging from 6.53 to 13.19%).

## Concluding remarks

The Serbian Health Care Consumers' Satisfaction Surveys between 2009 and 2015 revealed that most of the consumers of PHC centers were satisfied with the services, though there are differences with respect to gender, age, educational level, perceived HWB, health care expenses and unmet needs. During the observation period, the number of dissatisfied consumers increased and additional efforts will have to be undertaken to stop this trend. The evaluated data indicated that the most dissatisfied consumers were men, younger patients, patients with lowest educational level, patients with a bad perceived HWB and patients who had to pay the full price for GP visits, specialist visits and for drugs. Patients who missed or postponed visits because they could not pay for it, were also less likely to be satisfied with the offered PHC services.

Patients' satisfaction could be considered as a multidimensional quality indicator, depending on the structure and processes in healthcare delivery, as well as on the characteristics of the patients, their expectation from PHC etc. This multidimensionality is the reason why it is not so easy to explain the phenomenon of patient's satisfaction. Taking efforts to address the needs and expectations of consumers of different social, economic and demographic characteristics and to keep them satisfied with PHC could improve compliance with GP recommendations, thus ensuring better health outcomes and decreasing unnecessary health expenditures.

## Author contributions

All authors made substantial, direct and intellectual contribution to the work and approved it for publication.

### Conflict of interest statement

The authors declare that the research was conducted in the absence of any commercial or financial relationships that could be construed as a potential conflict of interest.

## References

[B1] Arsenijevic J. Pavlova M. Groot W. (2014). Out-of-pocket payments for public healthcare services by selected exempted groups in Serbia during the period of post-war healthcare reforms. Int. J. Health Plann. Manage. 29, 373–398. 10.1002/hpm.2188 23788401

[B2] Atanasova E. Pavlova M. Moutafova E. Rechel B. Groot W. (2013). Out-of-pocket payments for health care services in Bulgaria: financial burden and barrier to access. Eur. J. Public Health 23, 916–922. 10.1093/eurpub/cks169 23220626

[B3] Chaupain-Guillot S. Guillot O. (2015). Health system characteristics and unmet care needs in Europe: an analysis based on EU-SILC data. Eur. J. Health Econ. 16, 781–796. 10.1007/s10198-014-0629-x 25186072

[B4] Chue P. (2006). The relationship between patient satisfaction and treatment outcomes in schizophrenia. J. Psychopharmacol. 20, 38–56. 10.1177/1359786806071246 17046986

[B5] Davey A. Asprey A. Carter M. Campbell J. L. (2013). Trust, negotiation, and communication: young adults' experiences of primary care services. BMC Fam. Pract. 14:202. 10.1186/1471-2296-14-202 24373254 PMC3880848

[B6] Donabedian A. (2003). An Introduction to Quality Assurance in Health Care. New York, NY: Oxford University Press.

[B7] Eurostat (2016). Statistics Explained, Europe in Figures – Eurostat Yearbook. Available online at: http://ec.europa.eu/eurostat/statistics-explained/index.php/Eurostat_yearbook (Accessed January 4, 17)

[B8] Fenton J. J. Jerant A. F. Bertakis K. D. Franks P. (2012). The cost of satisfaction: a national study of patient satisfaction, health care utilization, expenditures, and mortality. Arch. Intern. Med. 172, 405–411. 10.1001/archinternmed.2011.1662 22331982

[B9] Garratt A. M. Solheim E. Danielsen K. (2008). National and Cross-National Surveys of Patient Experiences: A Structured Review. Oslo: Norwegian Knowledge Centre for the Health Services. 29320000

[B10] Glickman S. W. Boulding W. Manary M. Staelin R. Roe M. T. Wolosin R. J.. (2010). Patient satisfaction and its relationship with clinical quality and inpatient mortality in acute myocardial infarction. Circulation 3, 188–195. 10.1161/circoutcomes.109.900597 20179265

[B11] Health Consumer Powerhouse (2017). Euro Health Consumer Index 2016. Stockholm: The Health Consumer Powerhouse Ltd Available online at: http://www.healthpowerhouse.com/publications/euro-health-consumer-index-2016/ (Accessed February 7, 17).

[B12] Hinchcliff R. Greenfield D. Braithwaite J. (2014). Is it worth engaging in multi-stakeholder health services research collaborations? Reflections on key benefits, challenges and enabling mechanisms. Int. J. Qual. Health Care 26, 124–128. 10.1093/intqhc/mzu009 24519121

[B13] Jakovljevic M. B. Djordjevic N. Jurisevic M. Jankovic S. (2015). Evolution of the Serbian pharmaceutical market alongside socioeconomic transition. Expert Rev. Pharmacoecon. Outcomes Res. 15, 521–530. 10.1586/14737167.2015.1003044 25592856

[B14] Jakovljevic M. B. Vukovic M. Fontanesi J. (2016a). Life expectancy and health expenditure evolution in Eastern Europe - DiD and DEA analysis. Expert Rev. Pharmacoecono. Outcomes Res. 16, 537–546. 10.1586/14737167.2016.1125293 26606654

[B15] Jakovljevic M. Vukovic M. Chen C. C. Antunovic M. Dragojevic-Simic V. Velickovic-Radovanovic R.. (2016b). Do health reforms impact cost consciousness of health care professionals? Results from a nation-wide survey in the Balkans. Balkan Med. J. 33, 8–17. 10.5152/balkanmedj.2015.15869 26966613 PMC4767315

[B16] Jankovic J. Simic S. (2012). The association of demographic and socioeconomic determinants and self-perceived health. Serbian Arch. Med. 140, 77–83. 10.2298/sarh1202077j 22462352

[B17] Kontopantelis E. Roland M. Reeves D. (2010). Patient experience of access to primary care: identification of predictors in a national patient survey. BMC Fam. Pract. 11:61. 10.1186/1471-2296-11-61 20799981 PMC2936332

[B18] Kosanovic R. Andelski H. (2015). Basic directions of development health insurance in the Republic of Serbia (1922-2014). Zdravstvena zastita 3, 48–70 [Serbian].

[B19] Lim S. S. Allen K. Bhutta Z. A. Dandona L. Forouzanfar M. H. Fullman N. (2016). Measuring the health-related sustainable development goals in 188 countries: a baseline analysis from the Global Burden of Disease Study 2015. Lancet 388, 1813–1850. 10.1016/S0140-6736(16)31467-2 27665228 PMC5055583

[B20] Linder-Pelz S. (1982). Toward a theory of patient satisfaction. Soc. Sci. Med. 16, 577–582. 10.1016/0277-9536(82)90311-2 7100990

[B21] Ministry of Health of the Republic of Serbia (2005). Health Care Law. Official Gazette of the Republic of Serbia 107/05, 72/09, 88/10, 99/10, 57/11, 119/12, 45/13, 93/14, 96/15 and 106/15. Available online at: http://www.paragraf.rs/propisi/zakon_o_zdravstvenoj_zastiti.html (Accessed February 5, 17). [Serbian]

[B22] Ministry of Health of the Republic of Serbia (2006). Decree on the Health Care Institutions Network Plan. Official Gazette of the Republic of Serbia 42/06, 119/07, 84/08, 71/09, 85/09, 24/10, 6/12, 37/12, 8/14 and 92/15. Available online at: http://paragraf.rs/propisi/uredba_o_planu_mreze_zdravstvenih_ustanova.html (Accessed November 29, 16). [Serbian]

[B23] National Health Service Corporation (2010). Feeling Better? Improving Patient Experience in Hospital. London: The NHS Conferedation Available online at: https://kaggle2.blob.core.windows.net/competitions/kaggle/3199/media/Feeling_better_Improving_patient_experience_in_hospital_Report.pdf (Accessed December 1, 16)

[B24] Rademakers J. Delnoij D. de Boer D. (2011). Structure, process or outcome: which contributes most to patients' overall assessment of healthcare quality? BMJ Qual. Saf. 20, 326–331. 10.1136/bmjqs.2010.042358 21339310

[B25] Radevic S. Kocic S. Jakovljevic M. (2016). Self-assessed health and socioeconomic inequalities in Serbia: data from 2013 National Health Survey. Front. Pharmacol. 7:140. 10.3389/fphar.2016.00140 27303301 PMC4881383

[B26] Rahmqvist M. Bara A. C. (2010). Patient characteristics and quality dimensions related to patient satisfaction. Int. J. Qual. Health Care 22, 86–92. 10.1093/intqhc/mzq009 20133477

[B27] Ros C. C. Groenewegen P. P. Delnoij D. M. J. (2000). All rights reserved, or can we just copy? Cost sharing arrangements and characteristics of health care systems. Health Policy 52, 1–13. 10.1016/S0168-8510(00)00065-8 10899641

[B28] Saltman R. B. Rico A. Boerma W. (eds.). (2006). Primary Care in the Driver's Seat? Organizational Reform in European Primary Care. Maidenhead: Open University Press.

[B29] Sándor J. Kósa K. Papp M. Fürjes G. Kőrösi L. Jakovljevic M.. (2016). Capitation-based financing hampers the provision of preventive services in primary health care. Front. Public Health 4:200. 10.3389/fpubh.2016.00200 27679797 PMC5020077

[B30] Santric Milicevic M. Bjegovic Mikanovic V. Terzic Supic Z. Vasic V. (2011). Competencies gap of management teams in primary health care. Eur. J. Public Health 21, 247–253. 10.1093/eurpub/ckq010 20215334

[B31] Simic S. Santric Milicevic M. Matejic B. Marinkovic J. Adams O. (2010). Do we have primary health care reform? The story of the Republic of Serbia. Health Policy 96, 160–169. 10.1016/j.healthpol.2010.01.015 20181406

[B32] Sipetic S. Bjegovic-Mikanovic V. Vlajinac H. Marinkovic J. Jankovic S. Terzic Z.. (2013). The burden of disease preventable by risk factor reduction in Serbia. Vojnosanitetski pregled 70, 445–451. 10.2298/VSP111024049S 23789282

[B33] Terzic Supic Z. Bjegovic V. Marinkovic J. Santric Milicevic M. Vasic V. (2010). Hospital management training and improvement in managerial skills: Serbian experience. Health Policy 96, 80–89. 10.1016/j.healthpol.2010.01.002 20116126

[B34] Tucker J. L. (2002). The moderators of patient satisfaction. J. Manag. Med. 16, 48–66. 10.1108/02689230210428625 12069351

[B35] Walshe K. Freeman T. (2002). Effectiveness of quality improvement: learning from evaluations. Qual. Saf. Health Care 11, 85–87. 10.1136/qhc.11.1.85 12078378 PMC1743560

[B36] World Health Organization (2008). The World Health Report 2008: Primary Health Care – Now More Than Ever. World Health Organisation Available online at: http://www.who.int/whr/2008/en/ (Accessed December 10, 16)

[B37] World Health Organization (2015). European Health for All database (HFA-DB). Available online at: http://data.euro.who.int/hfadb/ (Accessed December 2, 16).

